# Is sonoelastography a helpful method for evaluation of parotid tumors?

**DOI:** 10.1007/s00405-012-2255-5

**Published:** 2012-12-13

**Authors:** Małgorzata Wierzbicka, Jarosław Kałużny, Ewelina Szczepanek-Parulska, Adam Stangierski, Edyta Gurgul, Tomasz Kopeć, Marek Ruchała

**Affiliations:** 1 Department of Otolaryngology, Head and Neck Surgery, Poznan University of Medical Sciences, 49 Przybyszewskiego St., 60-355 Poznan, Poland; 2 Department of Endocrinology, Metabolism and Internal Medicine, Poznan University of Medical Sciences, 49 Przybyszewskiego St., 60-355 Poznan, Poland

**Keywords:** Ultrasound, Sonoelastography, Salivary glands, Parotid, Tumors, Diagnosis

## Abstract

Sonoelastography is a novel technique, useful in a noninvasive assessment of lesions in multiple organs. The aim of the study was to examine whether the combination of conventional ultrasonography (US) with sonoelastography might improve the reliability of parotid tumor evaluation. Fourty-three consecutive patients with parotid tumors were surgically treated at a single tertiary center at the Department of Otolaryngology, Head and Neck Surgery. The sample included 27 women and 16 men, aged 15–80 (the mean age = 54 years). The reference group constituted of 54 healthy volunteers. High resolution grayscale ultrasonography (US) was performed preoperatively using a 15 MHz linear array transducer. Elastograms (ES) were scored by the conventional Ueno 5-point scale from ES1 (blue-soft) to ES5 (the entire lesion and surrounding area shaded red-stiff). In addition, detailed stiffness values in kPa were collected. The group consisted of 33 patients with benign and 10 patients with malignant tumors. The mean stiffness value was 146.6 kPa in 10 malignant tumors (mostly ES4) and 88.7 kPa in 33 benign tumors (mostly ES2 and ES3). The differences in tissue stiffness between normal parotid parenchyma in the reference group and the mean value for all tumors in the examined group were statistically significant (*p* < 0.001), and so was the case with the differences between the benign and malignant tumors (*p* < 0.001). Low stiffness scores (ES1,2) were found in 2 malignant and 15 benign tumors while high scores (ES3,4) were found in 8 malignancies and 18 benign tumors. Sonoelastography overlapping elasticity to the grayscale images supports additional informations. Preferential selection of the lesions characterized by high stiffness (ES4) improves the differential diagnosis of parotid tumors but the large degree of uncertainty of this method should also be pointed out.

## Introduction

Modern high resolution grayscale ultrasound (US) is currently the first-line imaging technique for the evaluation of parotid gland lesions in some European countries. The firm preoperative differential diagnosis of salivary gland tumors allows a more precise planning of surgical interventions [1–3]. The algorithm for evaluation of the salivary glands depends on the patient’s baseline status. While histopathology is a “gold standard”, fine-needle aspiration cytology (FNAC) is the reference standard in routine clinical practice [4, 5] in many departments. Sonoelastography (SE) is a novel imaging option involving tissue stiffness assessment, since malignant tissues are generally stiffer than benign components [4–6]. So far, SE has proven to be useful in noninvasive differentiation of benign and malignant lesions in multiple organs [7–10]. However, little is known about the potential of this diagnostic modality in evaluation of parotid tumors. Hence, the aim of the study was to examine whether the combination of conventional US imaging with SE might improve the evaluation of parotid gland tumors and to determine which cut-off elasticity value would be the best for differentiation of benign and malignant lesions.

## Materials and methods

Prospective analysis of salivary gland tumors by the means of high resolution grayscale ultrasonography and real-time qualitative and quantitative sonoelastography was performed. The examined group consisted of 43 consecutive patients with parotid gland tumors, treated surgically at a single tertiary center, at the Department of Otolaryngology, Head and Neck Surgery at Poznan University of Medical Sciences. The sample composed of 27 women and 16 men, aged 15–80 years (the mean age = 54 years, median = 57 years).

Ultrasonography was performed using a 15 MHz linear array transducer. Parotid gland parenchyma in US images was described as normal (homogenous) or heterogeneous (with hypo- or hyperechogenic regions). The recorded US features of each nodule included: size, echostructure, shape (round, oval, lobulated) and boundary (well or ill-defined). Three diameters of each tumor were measured. The patients with deep lobe tumors were excluded from the analysis, because US imaging is not a sufficient approach in such cases and extra imaging is required. On US pleomorphic adenomas usually have sharp borders, lobulated contours, homogenous structure, poor vascularization and acoustic enhancement. Well-defined, regular shape, heterogeneous echostructure, strong acoustic enhancement and irregular vascularization are typical of Warthin tumors. Malignant lesions were found to be heterogeneous, ill-defined and irregularly shaped with irregular vascularity pattern.

**Fig. 1:**
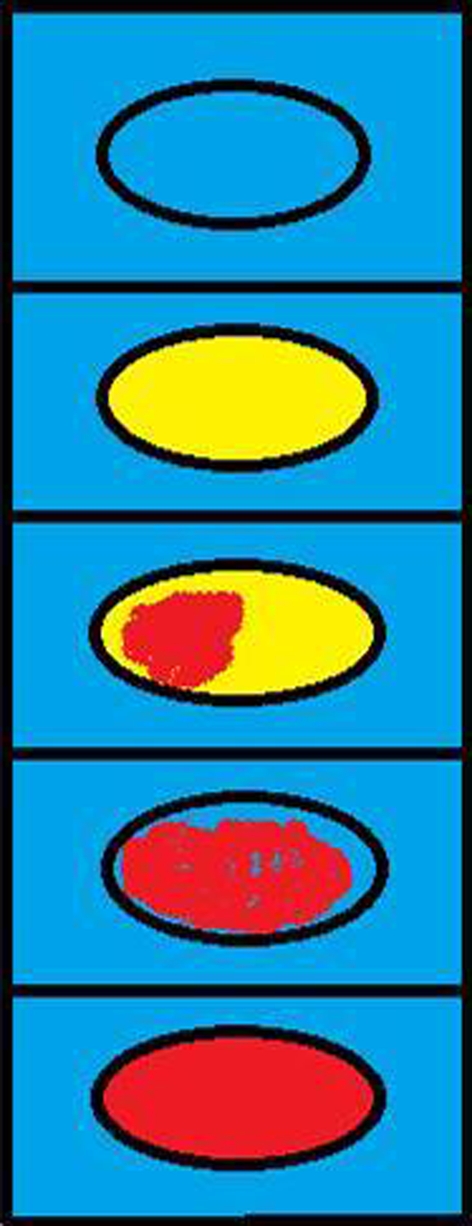
Ueno scale scheme: ES1 (*blue*-soft), ES2 (predominantly *green* to *yellow,* mostly soft), ES3 (less than 50 % of the area is shaded *red* and the rest of it is *yellow,* mildly stiff), ES4 (most of the lesion area is shaded *red* and the rest is *yellow,* mostly stiff) and ES5 (entire lesion area and surroundings are shaded *red*-stiff)

Sonoelastography was performed using AIXPLORER equipment by Supersonic Imagine with Super Linear SL-15-4 transducer. Transducer positioning was identical to that of conventional US. The monitor displayed elastograms and corresponding grayscale sonograms in real time. Elastograms were scored by the software upon conventional 5-point Ueno scale from ES1 (blue-soft), ES2 (predominantly green to yellow, mostly soft), ES3 (less than 50 % of the area is shaded red and the rest of it is yellow, mildly stiff), ES4 (most of the lesion area is shaded red and the rest is yellow mostly stiff) and ES5 (entire lesion area and surroundings are shaded red-stiff). The algorithm explaining quick color scale and how to distinguish particular grades from ES1 to ES5 is presented on the Fig. 1. In addition, the detailed stiffness values in kPa were collected. The minimal, maximal and mean values in four measurement points in different parts of the major salivary glands were calculated. The elastography values were obtained from specific sites of the mass: two from the center and two from the tumor periphery. All patients were operated on and histopathology was finally established.

Fifty-four healthy controls aged 43–78 years, (mean age = 60 years,) constituted the reference group for the SE values, not established so far. The subjects were recruited to the control group from the patients referred to the hospital due to other conditions than parotid gland tumor. Patients with a history or symptoms of any salivary gland pathology as well as those with a history of radiotherapy were excluded from the study. The elastography values were obtained from 4 points of sound parenchyma.

The main goal was to use the elastography as an adjunct approach to differentiate between benign versus malignant tumors. The elasticity of various benign tumors was determined as well. Statistical analysis was performed by means of Spearman’s rank correlation coefficient for the age analysis, Kruskal–Wallis Test for more than two variables (concerning histology) and Mann–Whitney *U* Test for two variables (benign/malignant tumor’s correlation).

## Results

The smallest diameter of the parotid lesion was 4 mm and the greatest one was 49 mm in the series. The volume of each tumor was assessed, with the mean value of 13.02 cm^3^ and median of 6.21 cm^3^.

The studied group composed of 33 patients with benign and 10 patients with malignant tumors. The final histopathological diagnosis of the tumors included: pleomorphic adenoma-23 cases, Warthin tumor-5 cases, monomorphic adenoma-2 cases, neurofibroma-1 case, cyst-1 case, basal cell adenoma-1 case, adenocarcinoma-3 cases, malignant lymphoma-2 cases, clarocellular carcinoma-1 case, nondifferentiated cancer-1case, squamous cell cancer-1 case, cancer from pleomorphic adenoma-1 case, and salivary duct cancer-1 case.

The sonographer suggested the presence of malignant neoplasms in 7 cases while the features considered typical of benign tumors were found in 33 cases (pleomorphic adenomas and Warthin tumor in 24 and 9 cases, respectively). In 3 patients the images were so unclear that a preliminary diagnosis of the lesion character was impossible, hence the patients were excluded from the ultrasonographic–histologic correlation. However, in these three cases elasticity was measured and analyzed.

The correlation of the results of US and histopathological examination was performed. Out of 33 tumors, initially characterized as benign in ultrasonography, in 30 (91 %) the diagnosis was confirmed by histopathology. In three cases the US result was false negative (1 nondifferentiated carcinoma, 2 malignant lymphomas). In 7 patients US features of malignant neoplasm were found. In 5 cases (71 %) malignant character was confirmed by histopathology. Three tumors of unclear sonographic picture got the diagnosis of: pleomorphic adenoma, carcinoma in pleomorphic adenoma and clarocellular cancer.

In addition, an effort was made to differentiate among various benign tumors. Histopathology confirmed the presence of pleomorphic adenoma in 21 out of 24 cases suspected in US (88 %). The other three tumors were diagnosed as: undifferentiated carcinoma, basal cell adenoma and cyst adenolymphoma. The presence of Warthin tumors was confirmed in 4 out of 9 cases (44 %) suspected, while other diagnoses included: pleomorphic adenoma, two lymphomas, branchiogenic cyst and monomorphic adenoma.

On histopathology, 7 malignant and 33 benign tumors in US images were true positive in 5 and 30 patients, respectively. Thus, the sensitivity of the malignant versus benign differentiation US was 93.8 % and the specificity was 62.5 %. In 30 out of 40 patients, the ultrasound result was true positive in predicting the tumor histopathology. The sensitivity of conventional US was 75 % and specificity was 70 %.

The histopathology of the tumors was confronted with the stiffness values in kPa. The SE of parotid gland parenchyma in 54 healthy controls who constituted the reference group and for 43 tumors are presented in Table 1. The mean stiffness of the lesion did not depend on the tumor size: dimension (*r*_S_ = 0.235) and volume (*r*_S_ = 0.228). The mean stiffness value in 10 malignant tumors was 146.6 kPa (ES4) and 88.7 kPa (ES2 and ES3) in 33 benign lesions. The differences in tissue stiffness between parotid gland parenchyma in the reference group and the mean value obtained for all tumors in the examined group were statistically significant (*p* < 0.001), and so were the differences between benign and malignant tumors (*p* < 0.001). The wide standard dispersion of deviations might be explained by the tumor heterogeneity: the center and periphery might differ significantly. Thus, elasticity measurement in a single tumor taken from the different points ranged from 5.0 kPa (min.) to 275.0 kPa (max.). The mean score showed the compromised values obtained from different regions and reflected the tumor specific mean stiffness.

**Table 1:** The histopathology of the surgical specimens and stiffness values in kPa in the examined group and healthy volunteers

	*N*	Tissue stiffness values in kPa			
		Mean	Minimum	Maximum	SD
All tumors	43	101.3	5.0	275.0	67.6
Malignant (all)	10	146.3	11.0	275.0	104.7
Benign (all)	33	88.7	5.0	237.5	48.0
Pleomorphic adenoma	23	89.9	5.0	237.5	43.8
Warthin tumor	5	80.9	30.0	158.5	52.3
Other benign lesions	5	84.8	28.0	189.0	57.0
Control group	54	26.0	11.0	47.5	8.7

*N* number of tumors, *SD* standard deviation

**Fig. 2:**
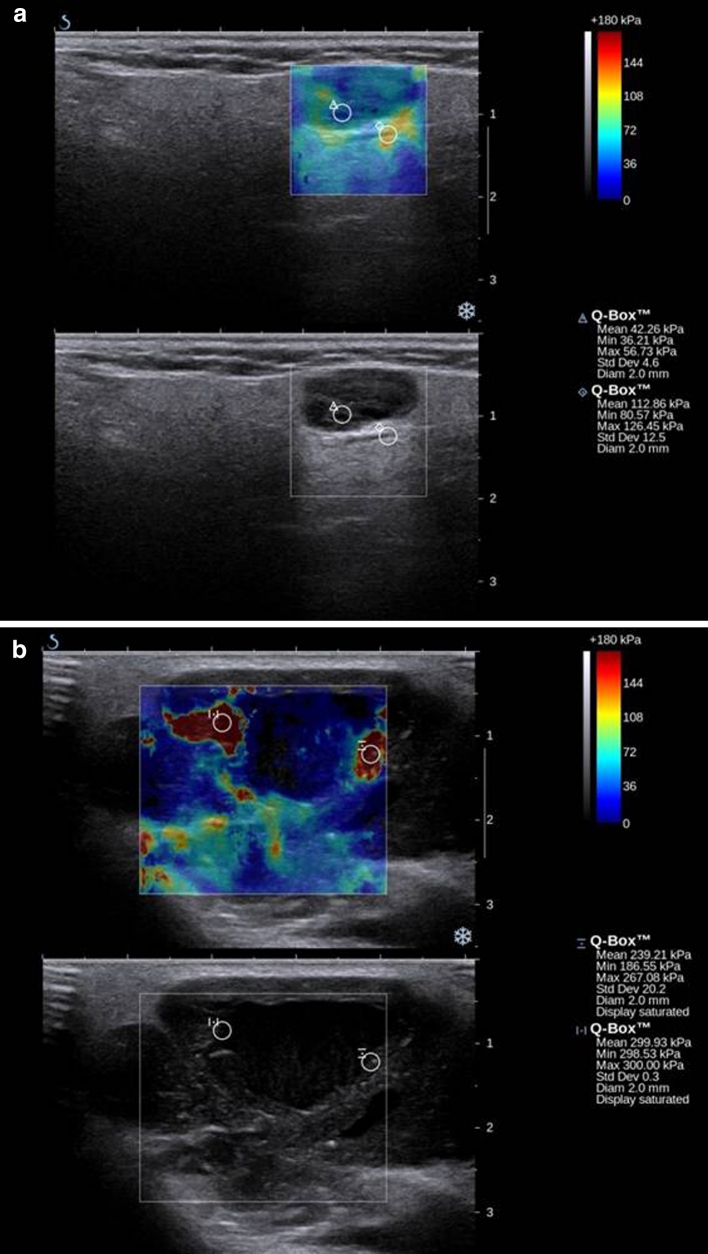
Figures representing Warthin tumor (**a**) and clarocellular cell cancer metastasis (**b**). Both lesions are smooth, round, hypoechogenic, with distal acoustic enhancement. Although the larger tumor appears more heterogeneous (**b**) than the smaller one (**a**), there was no suspicion of malignancy drawn by the ultrasonographist. Sonoelastography provided additional information about the nature of the lesions: the smaller lesion is plain *blue* and *green* (**a**) while the larger one (**b**) presented with *yellow* and *red* shading, indicating increased lesion stiffness

Lesion stiffness was visually graded on chromatic scale elastograms from ES 1 to 5 (low to high). The SE scoring of 43 salivary tumors is shown in Table 2. There were no lesions assessed as ES5, manifesting plain red shadowing. Parotid gland parenchyma in all 54 healthy controls that constituted the reference group, presented ES1 score. Out of the 43 lesions 8,9,10 and 16 tumors were classified as ES1, ES2, ES3 and ES4, respectively. Low elasticity (ES1,2) was found in 2 malignant and 15 benign tumors, while(ES3,4) 8 malignancies and 18 benign tumors were found to have high elasticity. The differences, however, were statistically insignificant. The sensitivity and specificity of SE was assessed regarding the potential of using this approach to differentiate between benign and malignant conditions. It was crucial to define the criteria of SE scoring to determine the stiffness characteristic exclusively for benign or malignant lesions. For this purpose, the values in kPa were measured and translated into the “quick” color scale. In this elastography option it is not necessary to calculate the kPa values. A single glance at the color allows the examiner to identify the tissue elasticity. The sensitivity and specificity were finally described on the basis of the convenient for everyday use Ueno 5-point scale in three modal options. (Table 3). The most important issue is to define the cut-off point to find out which of the ES stage would be considered the borderline between malignant and benign lesions: 2, 3 or 4. On the basis of analysis carried out in the examined group, the ES2 cut-off point means that score of two or lower indicates the benign character of the lesion with sensitivity of 89 %, but its specificity is low(45 %). Similarly, ES3 score indicates malignancy with sensitivity and specificity of 60 and 69.7 % while ES4 indicates sensitivity and specificity of 40 and 97 %, respectively. In the examined group, ES4 score was obtained for 4 out of 10 malignant tumors, 7 out of 23 pleomorphic adenomas and none of the Warthin tumors. The clinical example of the SE usefulness is shown in Fig. 2a and b.

**Table 2:** Elastography score (ES) of 43 salivary gland tumors

	*N*	Number of tumors according to elastography score (ES)			
		ES1	ES2	ES3	ES4
All tumors	43	8	9	10	16
Malignant (all)	10	1	1	2	6
Benign (all)	33	7	8	8	10
Pleomorphic adenoma	23	5	5	6	7
Warthin tumor	5	1	2	2	–
Other benign	5	1	1	2	1

*N* number of tumors

**Table 3:** Different percentages of sensitivity and specificity of elastographic evaluation, depending on the borderline elasticity (cut-off point)

Cut-off point	Sensitivity (%)	Specificity (%)
ES2	80.0	45.5
ES3	60.0	69.7
ES4	40.0	97.0

## Discussion

The specific characteristic of US images of malignant and benign tumors (especially pleomorphic adenomas and Warthin tumors) is well known and widely used for the purposes of differential diagnosis and surgical planning [1, 2, 11]. Real-time SE is a sonographic technique, involving tissue stiffness assessment, since malignant tissues are generally stiffer than benign components [4–6]. SE has been shown to differentiate malignant from benign lesions in the breast, prostate, liver and thyroid [12–14]. Few studies describing and validating this method in parotid gland pathology are available [15–17].

Our main purpose was to verify the usefulness of the SE in the differentiation of salivary glands’ focal lesions. Bhatia et al. [4, 5] and Dumitriu et al. [6] used real-time qualitative SE with free hand transducer compression. We used a modified technique, applied additional data and implemented a more elaborate SE scoring system. As it is the most important to define the criteria of SE scoring to determine the stiffness characteristic for benign or malignant lesions, the stiffness values in kPa were measured and translated into the “quick” color scale. We used the Ueno 5-point scale from blue ES1-soft to red color ES5-stiff. In the endpoint, this approach allowed for more objective lesion differentiation in the color scale for everyday use. There was also a different technical aspect. We used a novel ShearWave Elastography^®^ technique. It allows a completely objective, real-time and quantitative assessment of the tissue stiffness. Besides, there is no need to apply the transducer compressions, thus the assessment of lesion stiffness is more reliable. In this study, we were able to identify what key features might help in parotid tumors differentiation. The Warthin tumors were elastic (blue, green, small parts shaded yellow) and pleomorphic adenomas were predominantly elastic (green, yellow, less than half shaded red), however, such cases require careful analysis: 10 pleomorphic adenomas had ES 1/2 scores and 13 of them had ES 3/4 score. We found almost complete stiffness in six malignancies, and the mixed pattern of stiffness in two lymphomas and the renal metastatic tumor.

The values in kPa differed significantly for normal parenchyma, the mean value for all tumors and the mean value for malignant and benign lesions. The presence of cystic areas according to Dumitriu et al. [6] made the evaluation of the elasticity of the solid areas difficult by producing the typical color stratification pattern. However, this artifact could be useful in identifying small fluid areas, not apparent in US and distinguishing between a deeply hypoechogenic solid mass and a cyst. We confirmed this finding by presenting the identical clinical case when differentiating the Warthin tumor and clarocellular cancer metastasis in the Fig. 2.

Preliminary investigations suggested high potential of SE in predicting malignancy in thyroid nodules with the sensitivity of about 80–97 % and the specificity achieving 100 % [13, 15]. Bhatia et al. [4, 5] have seen, however, little benefit in the benign-malignant differentiation of parotid tumors. Dumitriu et al. [6] concluded that neither the internal structure nor the vascularization or sonoelastographic picture can identify a pleomorphic adenoma for certain and neglected the opportunity of differentiating between benign and malignant tumors by means of SE, as the pleomorphic adenomas also presented stiff components. Bhatia et al. [15] in their study on shear wave elastography presented practical aspects and potential pitfalls of the approach in diagnosing focal salivary gland lesions; the authors concluded that the potential role of elastography at this site is unclear and unsuitable for ruling out malignancy. Similarly, Westerland et al. [19] found the initial results to be disappointing. In our study, a statistically significant difference was found between benign and malignant tumor mean elasticity, measured objectively and quantitatively in kPa. Moreover, malignant tumors presented more extensive areas of stiffness. However, the very high standard deviation and range of the results partially confirm this skeptical point of view. The question arises how to deal with a dispersed standard deviation? Two opposite solutions are suggested. First, a smaller number of measurements should be performed and the tumor regions should be more carefully selected, i.e., only in hyperechogenic areas but the subjective, and not reproducible character of such a choice excludes this direction. On the contrary, inter-operator variations may be reduced if the medium value of more measurements will be taken into consideration.

The point is how to adopt the SE as the useful adjunct to the conventional US approach. Klintworth et al. [16] attempted to define and recognize any specific patterns of the distribution of stiff and soft areas. We would rather rely on more objective stiffness values, measured in kPa than on elastographic patterns. Thus, we propose to use elasticity values translated into the color scale. We suggest that the cut-off point at the level of ES4 provides the best differentiation between benign and malignant lesions. This approach ensures the 97 % SE specificity, though at the expense of sensitivity.

## Conclusions

The paper provides the description of sonoelastography application in preoperative assessment of benign and malignant parotid tumors. To elevate the specificity, we suggest the cut-off ES4 score to rule in the malignancies. The SE should be advocated for the simplicity of one-touch technique but the large degree of uncertainty should also be pointed out.
